# Transfer Tiling of Nanostructures for Large-Area Fabrication

**DOI:** 10.3390/mi9110569

**Published:** 2018-11-03

**Authors:** Jaekyoung Kim, Hyunsik Yoon

**Affiliations:** Department of Chemical Engineering, Seoul National University of Science & Technology, Seoul 01811, Korea; worud1200@seoultech.ac.kr

**Keywords:** tiling, nanostructures, nanoimprint, polymer, transfer

## Abstract

The fabrication of nanoscale patterns over a large area has been considered important but difficult, because there are few ways to satisfy both conditions. Previously, visually tolerable tiling (VTT) for fabricating nanopatterns for optical applications has been reported as a candidate for large area fabrication. The essence of VTT is the inevitable stitching of the nanoscale optical component, which is not seen by the naked eye if the boundary is very narrow while the tiles are overlapped. However, it had been difficult to control the shape of the spread of liquid prepolymers in the previous work, and there was room for the development of tiling. Here, we propose a method for transferring various shapes of tiles, which can be defined with a shadow mask. The method of using a transparent shadow mask can provide a wide process window, because it allows the spreading of a liquid prepolymer to be more easily controlled. We optimize the coating condition of a liquid prepolymer and the ultraviolet (UV) exposure time. Using this method, we can attach tiles of various shapes without a significant visible trace in the overlapped region.

## 1. Introduction

There is a growing demand for methods that can be implemented across large areas, as the need for micro- or nanoscale surface features increases [1,2,3,4,5,6,7,8,9,10]. One example is the fabrication of wire grid polarizers (WGPs) for use in display devices, such as liquid crystal displays (LCDs). Wire grid polarizers are arrays of metal lines of sub-100 nm width, and an optical component for polarizing incident lights. To fabricate the nanoscale structures for WGPs, e-beam lithography is necessary to prepare a master for nanoimprint lithography (NIL) or a mask for photolithography [11,12,13,14,15,16,17,18,19]. However, the process time for writing nanoscale patterns for a whole area in e-beam lithography is too long, and the photolithographic equipment to ensure sub-100 nm structures is significantly too expensive. A step-and-repeat process with a mask or a mold without stitching marks can be a candidate, due to the low cost and the large area fabrication of seamless WGPs; however, a perfect alignment in nanoscale cannot be achieved in a commercialized equipment. Previously, the Guo group reported a pioneering work, which named visually tolerable tiling (VTT) to fabricate large-area optical components, such as WPGs [5]. They demonstrated large-area nanostructures by overlapping the tiles in a small stamp onto a substrate. The key of the VTT is that the optical properties of the tiles should be identical in all directions, and that the boundary area should be narrow enough not to be seen by naked eyes. For example, anti-reflection properties with nano-domes are identical regardless of the positions of nanostructures under a sub-visible light wavelength, while microlens or microprism arrays should be aligned perfectly, because the refraction on the surfaces of microstructures can be different in different directions. In the case of WGPs, the optical properties are identical in all directions if the orientation of the lines on different tiles matches each other. The Guo group proved that the overlapped boundary is visually tolerable, although the nano-lines are not connected during the tiling procedure [5]. However, there still remains the need to control the spreading of liquid prepolymer, which is sensitive to manipulation during the pressing of a mold on a substrate. Additionally, the shape of the spreading was rounded, which is not suitable for being put together for fabrication in a large area. To resolve the problem, we propose a method to transfer shaped tiles using a transparent shadow mask. Rectangular tiles have the advantage of being able to attach several times, unlike the previous rounded ones. We use a two-step ultraviolet (UV)-exposure method. First, UV exposure is used to remove cured polymers from the undesired area, and second, the partially-cured prepolymer of a designed shape is attached to the target substrate. In this work, we compare the spreading ratio by controlling the film thickness of a liquid prepolymer and UV curing time. Finally, we demonstrate a tiling for the stitching of two tiles, and show that there is no visual difference in the boundary region.

## 2. Materials and Methods

**Figure 1:**
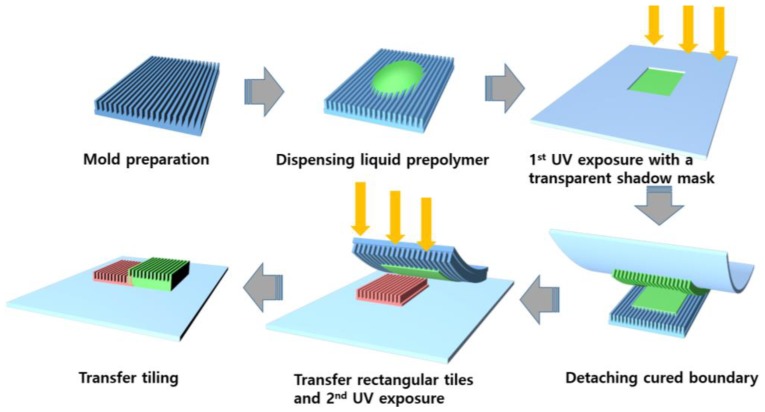
Schematic illustration of the experimental procedure.

In the experiment, we used two line-and-space masters of different sizes. One master was a sub-micron grating pattern, with a width of 800 nm, spacing at 400 nm, and a height of 200 nm. To prepare the master, we fabricated it by using conventional lithography and a dry etching process. From the master, we could prepare two types of replica molds. Elastomeric polydimethylsiloxane (PDMS) molds were prepared by pouring a mixture of prepolymer and a crosslinking agent in a 10:1 ratio, followed by thermal curing at 60 °C for 4 h. We also prepared a replica mold with polyurethane acrylate (PUA) prepolymer mixed with a photoinitiator (RM-3300, MCNet, Gyeonggi, Korea), by detaching it from the master after UV curing. The difference between the PDMS and PUA molds is the modulus and the surface energy. Due to the relatively low aspect ratio of the grating pattern, PDMS can be used without lateral collapse issues. The other master is a sub-100 nm pattern for WGPs. We purchased the commercially-available WGP films from Edmond Optics (Barrington, NJ, USA). The pattern width was 80 nm, the spacing was 60 nm, and the height was 140 nm. When we use sub-100 nm nanostructures with a wire-grid polarizer master, we use PUA as the replica mold material, because the structure is difficult to replicate with PDMS due to the lateral collapse problem, resulting from a low modulus [19]. The experimental procedure of the transfer tiling is shown in Figure 1. After the preparation of the mold (PDMS or PUA), we dropped a liquid prepolymer onto the master. In this experiment, we used a mixture of PUA prepolymer with a photoinitiator to realize nanoscale patterns by UV nanoimprint lithography. We noted that the PUA can do the self-replication, as previously reported [19]. After preparing a transparent polyethylene terephthalate (PET) film, with a rectangular frame cut by a machine (Silhouette Cameo, Orem, UT, USA), we place the PET shadow mask onto the surface of the liquid prepolymer directly. As opposed to the original usage of a shadow mask to screen a metal deposition in the undesired region, UV light transmits through the PET film and crosslinks the prepolymer, which is sandwiched between the PET film and the mold, while the prepolymers in the rectangular area (which are open to the air) are not crosslinked, due to the inhibition of crosslinking by the oxygen [18]. After UV exposure, we removed the cured PUA bonded on the PET frame from the mold, and the partially-cured prepolymer tiles of a rectangular shape remained on the mold. Then, we placed this tile next to the pre-defined tiles, and exposed them to UV light, in order to crosslink the partially-cured prepolymer. At last, we could obtain two tiles attached to the same substrate, as shown in the last step of Figure 1.

## 3. Results and Discussion

**Figure 2:**
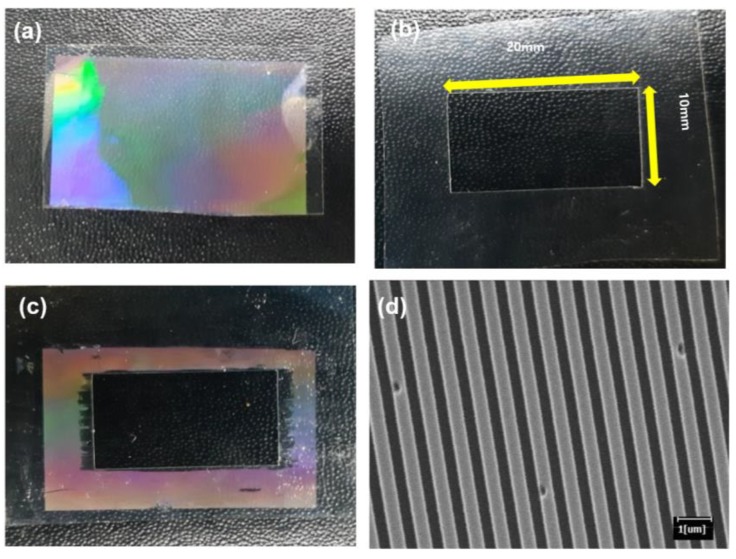
(**a**) A picture of a polyurethane acrylate (PUA) mold, replicated from a master. (**b**) Transparent shadow mask, prepared by a conventional cutting machine. (**c**) Shadow mask, after curing the prepolymer outside of the boundary. (**d**) Scanning electron microscopic (SEM) image of the nanoscale pattern.

The picture of a replicated mold is shown in Figure 2a. After the preparation of the transparent shadow by a cutting machine (Figure 2b), we detached the cured polymer from the undesired area after UV exposure, as shown in Figure 2c. The transparent shadow mask is similar to the metallic shadow mask used in the physical deposition, because the undesired region is removed by the shadow mask. The nanoscale pattern of the nanoscale pattern (800 nm in width, 400 nm in spacing), fabricated by PUA, is shown in Figure 2d. To define the tile shape, we tried to attach the tape in the undesired region, followed by removing the unnecessary region, as shown in Figure S1. When we use a PDMS mold (Figure S2a), we could define the boundary by the tape, because there is no residue after detaching the tape from the PDMS mold (Figure S2b). When we use a PUA mold for better resolution in nanoscale patterning, which is due to its higher Young’s modulus, the residue of the tape remains after detaching the tape from the mold (Figure S2c,d). For this reason, we propose a two-step UV exposure method, in order to define the shape of the tile and the process of attachment to the substrate by using a transparent shadow mask.

**Figure 3:**
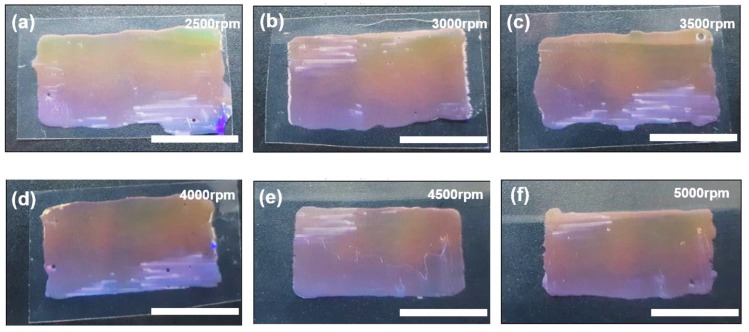
Pictures of the tiles, changing the speed of the spin coating: (**a**) 2500× rpm, (**b**) 3000× rpm, (**c**) 3500× rpm, (**d**) 4000× rpm, (**e**) 4500× rpm, and (**f**) 5000× rpm. The scale bars represent 1 cm.

To investigate the degree of spreading of liquid prepolymer in the defined region, we controlled the rotation speed of the spin coating and UV exposure time. We coated the liquid prepolymer at 2500 to 5000 rotations per minute (rpm) to vary the coating thickness. As shown in Figure 3, the liquid in the defined region spread more when the spinning speed was slow. Additionally, the liquid spreading prohibits the definition of the tile shape, because the spreading direction is random as the tiles are sandwiched on a substrate. When we reduced the thickness (via a higher spinning speed), we could obtain rectangular tiles, as shown in Figure 3e,f. Additionally, we controlled the UV exposure time for curing to control the spreading of the liquid prepolymer. Although oxygen prohibits the crosslinking the UV-curable prepolymer, the prepolymer becomes viscous due to the partial crosslinking.

**Figure 4:**
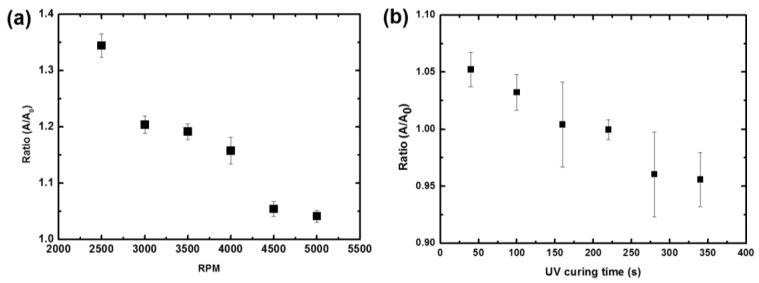
(**a**) A graph showing the ratio of the spreading area, compared to the design area. The ratio approaches unity when the spinning speed is high. (**b**) Ratio dependence on the ultraviolet (UV) curing time.

The graph of the ratio of the spreading area (A), compared to the designed area (A_0_ = 20 mm × 10 mm), is shown in Figure 4a. The ratio approaches unity when the rotation speed is higher than 4500× rpm. This means that the spreading of the liquid prepolymer depends on the film thickness, although the films are partially cured. Figure 4b shows the effect of the UV partial curing time. When the partial curing time is increased, the liquid prepolymer becomes more viscous, and the area change is not severe. We note that the spinning speed is fixed at 4500× rpm when we change the UV curing time. From the optimized rotational speed condition, we transfer the tiles next to a previously patterned tile.

**Figure 5:**
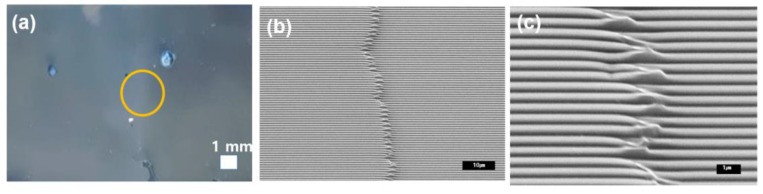
(**a**) A picture of the tiling next to the defined structures. The circle represents the boundary between the two tiles. (**b**) An SEM image of the boundary region. (**c**) A magnified image of (**b**). The defects (merged lines) cover 2~3 µm, although the boundary is almost invisible.

To further distinguish the visual difference, we deposited an aluminum layer (20 nm) onto the transferred tiles. The image of the two tiles, overlapped in the center, is shown in Figure 5a. We attached the second tile to the left of the previous tile. The boundary is overlapped by the second tile. It is not visually different to the naked eye, as shown in Figure 5a. The scanning electron microscopic (SEM) image of the boundary is shown in Figure 5b,c. The second tiles overlapped to the left, and the sub-100 nm patterns were fabricated with a high aspect ratio.

**Figure 6:**
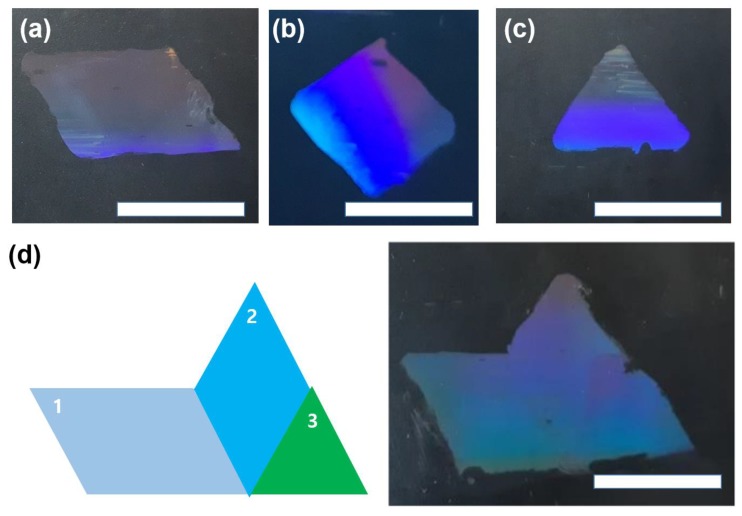
Pictures of the tiles, with the shape of a (**a**) parallelogram, (**b**) diamond, and (**c**) triangle, as well as (**d**) an assembled pattern with the parallelogram, diamond, and triangle-shaped tiles. The scale bars represent 1 cm.

Another advantage of transfer tiling is the possibility of making various shapes on demand. The shape of tiles can be parallelogram (Figure 6a), diamond (Figure 6b), or triangular (Figure 6c). As shown in Figure 6d, the tiling can show a variety of shapes, even with tapered tiles. The freedom of the tile shapes or sizes can be an advantage for fabrication nanoscale patterns in a large area or patterned substrates.

## 4. Conclusions

In this work, we propose a transfer method for various shapes of tiles for the purpose of fabricating optical films in a large area. A transparent shadow mask, made from a PET frame prepared by a conventional cutting machine, can define the shapes of the tiles. We control the spreading of liquid prepolymer by controlling the spinning speed and optimizing the conditions for making tiles. We demonstrate that the tiles overlapped, but not to such a degree as would be visible to the naked eye. In addition, the shapes can be a rectangle, parallelogram, or triangle, which gives us the freedom of design. Transfer tiling can offer a methodology for preparing large-area optical films originating from nanoscale textures, such as anti-reflection and wire-grid polarizers.

## Supplementary Materials

The following are available online at http://www.mdpi.com/2072-666X/9/11/569/s1. Figure S1: Experimental procedure using tape to define the tile shapes; Figure S2: (a) A PDMS mold, with tape to remove the outside of the boundary. (b) The prepolymer, clearly removed by detaching the tape. (c) The PUA mold, adhered using tape. (d) The sticky residues remaining on the PUA mold.
