# Supplementary material for: Transfer Tiling of Nanostructures for Large-Area Fabrication
Source: Micromachines (Basel). 2018 Nov 3;9(11):569. doi: 10.3390/mi9110569 (PMC6266267; doi:10.3390/mi9110569)
Supplement: Supplementary file 1 [file micromachines-09-00569-s001.pdf]

Supplementary Materials: Transfer Tiling of Nanostructures for Large-Area Fabrication

Jaekyoung Kim, and Hyunsik Yoon

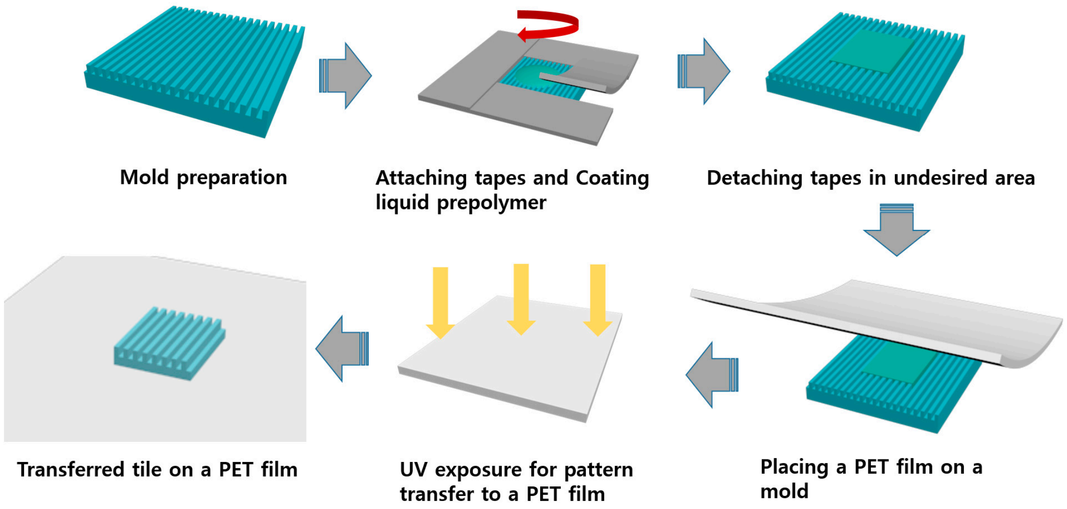

Figure S1. Experimental procedure using tape to define the tile shapes.

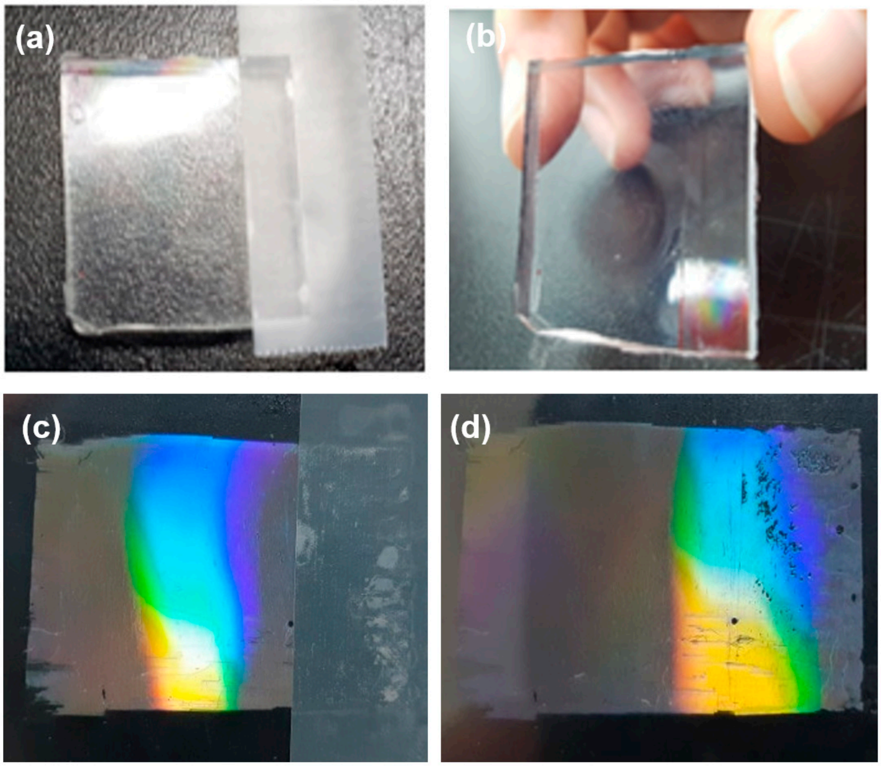

Figure S2. (**a**) A PDMS mold, with tape to remove the outside of the boundary. (**b**) The prepolymer, clearly removed by detaching the tape. (**c**) The PUA mold, adhered using tape. (**d**) The sticky residues remaining on the PUA mold.
